# Associations between 47 anthropometric markers derived from a body scanner and relative fat-free mass in a population-based study

**DOI:** 10.1186/s12889-024-18611-w

**Published:** 2024-04-18

**Authors:** Maximilian Dietzmann, Dörte Radke, Marcello RP Markus, Mats Wiese, Henry Völzke, Stephan B. Felix, Marcus Dörr, Martin Bahls, Till Ittermann

**Affiliations:** 1 Institute for Community Medicine, University Medicine Greifswald, Walther Rathenau Str. 48, D-17475 Greifswald, Germany; 2 German Centre for Cardiovascular Research (DZHK) partner site Greifswald, Greifswald, Germany; 3 Department of Internal Medicine B, University Medicine Greifswald, Greifswald, Germany; 4 Department of Internal Medicine A, University Medicine Greifswald, Greifswald, Germany

**Keywords:** Body surface scan, anthropometric parameters, Fat free mass, Cardiovascular disease, Epidemiology, Population-based study

## Abstract

**Background:**

Low relative fat free mass (FFM) is associated with a greater risk of chronic diseases and mortality. Unfortunately, FFM is currently not being measured regularly to allow for individuals therapy.

**Objective:**

One reason why FFM is not being used may be related to additional equipment and resources, thus we aimed to identify easily accessible anthropometric markers related with FFM.

**Materials and methods:**

We analyzed data of 1,593 individuals (784 women; 49.2%, age range 28–88 years) enrolled in the population-based Study of Health in Pomerania (SHIP-TREND 1). Forty-seven anthropometric markers were derived from a 3D optical body-scanner. FFM was assessed by bioelectrical impedance analysis (FFM_BIA_) or air displacement plethysmography (FFM_ADP_). In sex-stratified linear regression models, FFM was regressed on anthropometric measurements adjusted for body height and age. Anthropometric markers were ranked according to the coefficient of determination (R^2^) derived from these regression models.

**Results:**

Circumferences of high hip, belly, middle hip, waist and high waist showed the strongest inverse associations with FFM. These relations were stronger in females than in males. Associations of anthropometric markers with FFM_APD_ were greater compared to FFM_BIA_.

**Conclusion:**

Anthropometric measures were more strongly associated with FFM_ADP_ compared to FFM_BIA_. Anthropometric markers like circumferences of the high or middle hip, belly or waist may be appropriate surrogates for FFM to aid in individualized therapy. Given that the identified markers are representative of visceral adipose tissue, the connection between whole body strength as surrogate for FFM and fat mass should be explored in more detail.

**Supplementary Information:**

The online version contains supplementary material available at 10.1186/s12889-024-18611-w.

## Introduction

Due to the ageing population in Western societies, there is a growing interest of assessing body composition in the clinical setting. Relative fat-free mass (FFM, described as % of total body weight) is directly related to cardiorespiratory fitness [1] and inversely associated with all-cause, cardiovascular, and cancer mortality [2–4]. Low FFM is also an inherent characteristic of malnutrition and sarcopenia [5, 6]. Physical disability and a reduced functional capacity are also related to a lack of FFM [7, 8]. In addition, a small FFM is associated with a higher prevalence of cardiovascular risk factors like hypertension [9], obesity or diabetes [3]. Overall, FFM is of growing clinical interest and needs to be assessed in clinical practice to aid in individualized therapy.

FFM can be precisely determined by imaging methods such as quantitative magnetic resonance imaging (MRI), dual-energy X-ray absorption (DXA), ordinary MRI or computed tomography scans [10, 11]. However, these methods are commonly time consuming, rely on large and partly expensive stationary hardware and often go hand in hand with radiation exposure. Considering the aforementioned limitations, these procedures are inconvenient or infeasible for practical reasons in the clinical setting and in large epidemiological studies.

Bioelectrical impedance analysis (BIA) can be considered an indirect, yet radiation free, time-efficient and portable alternative for FFM estimation [10]. Nonetheless, BIA has some limitations. BIA requires some assumptions like constant hydration status [12] and this technology may not be used in patients with implanted cardiac devices [13]. Albeit, FFM measurements based on BIA (FFM_BIA_) has substantial variability on the individual level, satisfactory reliability has been reported on a population level [14–16].

Air displacement plethysmography (ADP) may also be used to quickly assess FFM (FFM_ADP_) [17, 18]. This method is also radiation-free and time-efficient, though not very portable. Various studies have shown high test-retest-reliability [19] of ADP in the assessment of body composition and good validity compared to DXA or BIA [20, 21].

Since the assessment of FFM, independent of method, is currently hampered by time and costs, we aimed to identify easily accessible anthropometric markers. Automatic three-dimensional optical body scans are a reliable and repeatable method to identify potential anthropometric biomarkers [22]. This process is completely radiation free and safe. Importantly, this method agrees with the reference method of manual measurements fulfilling WHO criteria [22–24].

Previous studies with relatively small sample sizes reported associations between three-dimensional body surface anthropometrics and data derived from BIA [23, 25] or ADP [26]. However, the study population was rather small and consisted almost exclusively of healthy men or even athletes. A systematic review summarizing associations of body scan markers with FFM_ADP_ and FFM_BIA_ [27] reported that body scanners had a high degree of accuracy and reliability. The major limitations of the previous studies were the small sample sizes as well as the homogenous study populations which we tried to address in our analysis.

The rationale for this study was to identify easily accessible anthropometric markers of FFM to aid in individualized therapy. A prime example could be the use of resistance training to either reduce the loss or even gain fat-free mass (i.e. skeletal muscle). Especially in rural and/or economically not very successful regions of the world, the above mentioned technologies may not be available. Yet, inhabitants of these regions may also suffer from cardiovascular risk factors and/or age-induced sarcopenia. This study aims to identify potential surrogates of FFM by investigating associations of automatic body scanner-derived anthropometric measurements with FFM as determined by BIA and ADP in a large-population-based sample including 1,593 individuals aged 28–88 years to provide alternatives. Since individuals with higher BMI have also higher FFM, we decided to use FFM normalized for total body weight as an outcome to not underestimate the effect of obesity on the relation between anthropometric markers and FFM.

## Materials and methods

### Study population

**Fig. 1:**
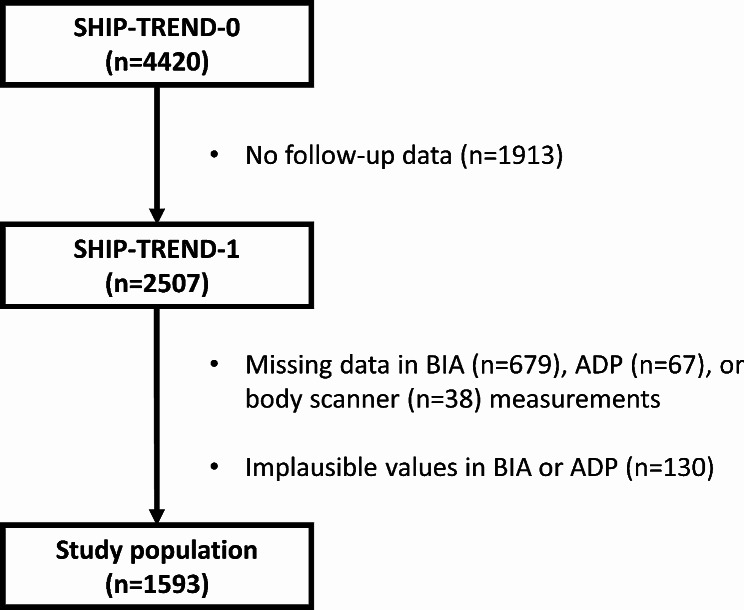
Flow chart on the selection process of the study population

The Study of Health in Pomerania (SHIP), conducted by the University Medicine Greifswald, has been designed as a population-based project assessing common risk factors and subclinical disorders in the adult population [28]. For the present analyses, we used data from the SHIP-TREND-1 cohort. SHIP-TREND-1 is the first follow-up of the population-based SHIP-TREND study, in which 2,507 individuals were examined between 2016 and 2019. We excluded 679 participants without BIA and 67 participants without ADP measurements. In addition, we did not include 38 participants with missing body scanner values and 130 participants with implausible BIA or ADP measurements resulting in a study population of 1,593 individuals (Fig. 1).

### Anthropometric measurements

Manual anthropometric measurements included height, weight, waist- and hip circumference. Weight was measured to the nearest 0.1 kg in light clothing and without shoes using standard digital scales. Waist circumference (WC) was measured to the nearest 0.1 cm using an inelastic tape midway between the lower rib margin and the iliac crest in the horizontal plane with the subject standing comfortably with weight distributed evenly on both feet. Hip circumference (HC) was measured to the nearest 0.1 cm using an inelastic tape midway between the iliac crest and the most lateral (sideways) protruding points of the greater trochanter. Waist-to-hip ratio (WHR) was calculated as WC/HC. Body mass index (BMI) was calculated as weight [kg] divided by height to the square [m^2^].

#### 3D optical body scan

Anthropometric data was measured with a three-dimensional optical body scanner *(VITUS Smart XXL, Vitronic, Wiesbaden, Germany)* driven by the software *AnthroScan Professional (Version 3.0.7, Human Solutions GmbH, Kaiserslautern, Germany).* This measuring technique is based on the optical triangulation process using four lasers and eight cameras, to date one of the most precise method of contactless capture of body shape. The apparatus allows the observer to receive deformation-free measurements, since neither the lasers nor the sensors enter into any physical contact with the study participant [29, 30].

The measurement results in a detailed three-dimensional image of the participants’ body surface and extracts a large number of standard anthropometric markers, including circumferences, lengths, distances, areas, volumes and their ratios within 10–15 s. After exclusion of markers which were highly associated with other anthropometric markers (e.g. different height measurements) and markers which cannot be measured standardized in clinical practice (e.g. distance back to the wall), we used 47 standard anthropometric markers for the present analyses (Supplementary Tables 1 and 2).

While being scanned participants wore underwear and a fabric head cap in order to reduce measurement error due to hair volume. After measuring body height twice (sitting and standing position) a so-called standard-scan allowed measuring aforementioned anthropometric markers in an upright standing position. More details on the assessment can be found in the supplement.

#### Bioelectrical impedance analysis

BIA was performed using a multifrequency *Nutriguard-M* device *(Data Input GmbH, Pöcking, Germany)* and the *NutriPlus* software *(Version 5.4.1, Data Input GmbH, Pöcking, Germany)*. R (resistance) and X_c_ (reactance) were measured applying electric currents of 800 mA at 5, 50, and 100 kHz following the manufacturer’s instructions [31, 32]. Source and sensor electrodes were placed on the dorsum of hand, wrist, ankle and dorsum of foot of the right side of the body with participants in supine position [33]. Absolute FFM was calculated within the software. Relative FFM was calculated in % by dividing absolute FFM by total body weight and then multiplying by 100. The BIA examination took place on a different date than the core examination. For more details, see supplement.

#### Air displacement plethysmography

Air displacement plethysmography was performed using a *BOD POD*^®^*(COSMED Deutschland GmbH, Werneck, Germany)* device according to the manufacturer’s recommendation *(COSMED Deutschland GmbH, Werneck, Germany)*. This method is a densitometric measuring method and can be used to determine body volume, body density, lung volume and fat mass. Absolute FFM was calculated by subtracting body weight and fat mass. Relative FFM was calculated in % by dividing absolute FFM by total body weight and then multiplying by 100.

### Other clinical measurements

Non-fasting blood samples were taken and serum levels of low density lipoprotein (LDL-C) cholesterol, high density lipoprotein (HDL-C) cholesterol, triglycerides, serum glucose and glycated hemoglobin (HbA1c) were assessed by a *Dimension Vista 500* analytical system *(Siemens AG, Erlangen, Germany)*. Blood pressure was measured using a *HEM 705CP* device *(Omron Corporation, Tokyo, Japan)*. Arterial hypertension was defined as systolic blood pressure ≥ 140 mmHg or diastolic blood pressure ≥ 90 mmHg or antihypertensive treatment (Anatomical Therapeutic Chemical Classification System (ATC) codes C02, C03, C04, C07, C08 or C09A). Known or newly detected diabetes mellitus was defined as self-reported physician’s diagnosis or intake of glucose-lowering drugs (ATC code A10) or HbA1c concentrations of ≥ 6.5% (≥ 47.54 mmol/mol) or non-fasting serum glucose > 11.1 mmol/l.

### Statistical analysis

Characteristics of the study population are reported stratified by sex as absolute numbers and percentages for categorical data and as median, 25th, and 75th percentiles for continuous data. Since individuals with greater absolute FFM also have a higher fat mass, FFM is positively related with cardiovascular risk. Hence, we used relative FFM as the outcome in our analysis [4]. Anthropometric markers were associated with FFM by sex-stratified linear regression models adjusted for age and body height. Since men and women show significant differences with regards to cardiometabolic biomarkers [34], body fat distribution [35] and body composition [36] we decided that a sex-stratified analysis is warranted. To make the effect sizes of the different anthropometric markers comparable, all anthropometric markers were z-standardized. Stratified by sex and measurement technique, anthropometric markers were ranked based on the coefficients of determination (R^2^). As a result, we derived for each variable a β-coefficient, a 95%-confidence interval, a R^2^, a *p* value, and a -log *p* value. For each sex the β-coefficients and 95%-confidence intervals for the ten variables with the highest R^2^ were plotted. Furthermore, we created a heatmap, in which all body scanner variables were included that were one of the ten variables most strongly associated with FFM_BIA_ or FFM_ADP_ in men or women. In addition, we conducted a sex-stratified random forest regression, in which we included all 47 body scan markers together with age and height as explanatory variables and the respective FFM as outcome. Before applying the random forest, the data was randomly split into equally-sized training and test datasets. Before applying the final models, we optimized the hyper-parameters “number of iterations” (numit) and “number of variables to randomly investigate at each iteration” (numvars) by minimizing the out of bag (oob) and validation errors as described by Schonlau et al. [37]. Afterwards, the final random forest models with the optimized hyper-parameters were calculated and the most important variables for each setting were plotted. All statistical analyses were performed using *Stata 18.0 (Stata Corporation, College Station, TX, USA)*.

## Results

### Characteristics of the study population

The study sample included 1,593 individuals (49.2% females, 28–88 years). Males were slightly older and had a higher BMI than females (Table 1). Independent of method, FFM was higher in males than in females.

**Table 1:** Characteristics of the study population stratified by sex

	Males (*n* = 809)	Females (*n* = 784)
Age; years	57 (47; 67)	55 (46; 65)
Body mass index; kg/m^2^	27.9 (25.7; 30.7)	26.6 (23.4; 30.3)
Waist circumference; cm	101 (93; 109)	88 (80; 99)
Hip circumference; cm	100 (95; 106)	102 (95; 111)
Fat-free mass (BIA); %	72.7 (69.0; 77.1)	63.3 (58.5; 68.1)
Fat-free mass (ADP); %	70.3 (65.4; 75.8)	59.6 (54.0; 65.9)
Alcohol consumption; g/day	9.2 (3.0; 20.0)	3.3 (1.0; 7.9)
Smoking status Never Former Current	223 (27.6%) 446 (55.1%) 140 (17.3%)	329 (42.0%) 314 (40.1%) 141 (18.0%)
Sports score according to Baecke [55]	2.4 (0.0; 5.1)	2.3 (0.0; 4.2)
Systolic blood pressure; mmHg	128 (121; 137)	120 (111; 129)
Diastolic blood pressure; mmHg	76 (70; 82)	73 (67; 79)
Hypertension	421 (52.0%)	289 (36.9%)
LDL-cholesterol; mmol/l	3.29 (2.63; 3.87)	3.39 (2.8; 4.05)
HDL-cholesterol; mmol/L	1.31 (1.08; 1.53)	1.64 (1.38; 1.96)
Triglycerides; mmol/L	1.38 (0.92; 2.02)	1.08 (0.76; 1.62)
Blood glucose; mmol/L	5.5 (5.0; 6.0)	5.1 (4.8; 5.5)
HbA1c; %	5.5 (5.3; 5.8)	5.5 (5.3; 5.7)
Type 2 diabetes	78 (9.6%)	48 (6.1%)

Continuous data are expressed by median, 25th, and 75th percentile; categorical data by absolute numbers and percentages (LDL = low-density lipoprotein, HDL = high-density lipoprotein, HbA1c = glycated hemoglobin)

### Association of FFM assessed by BIA and ADP

**Fig. 2:**
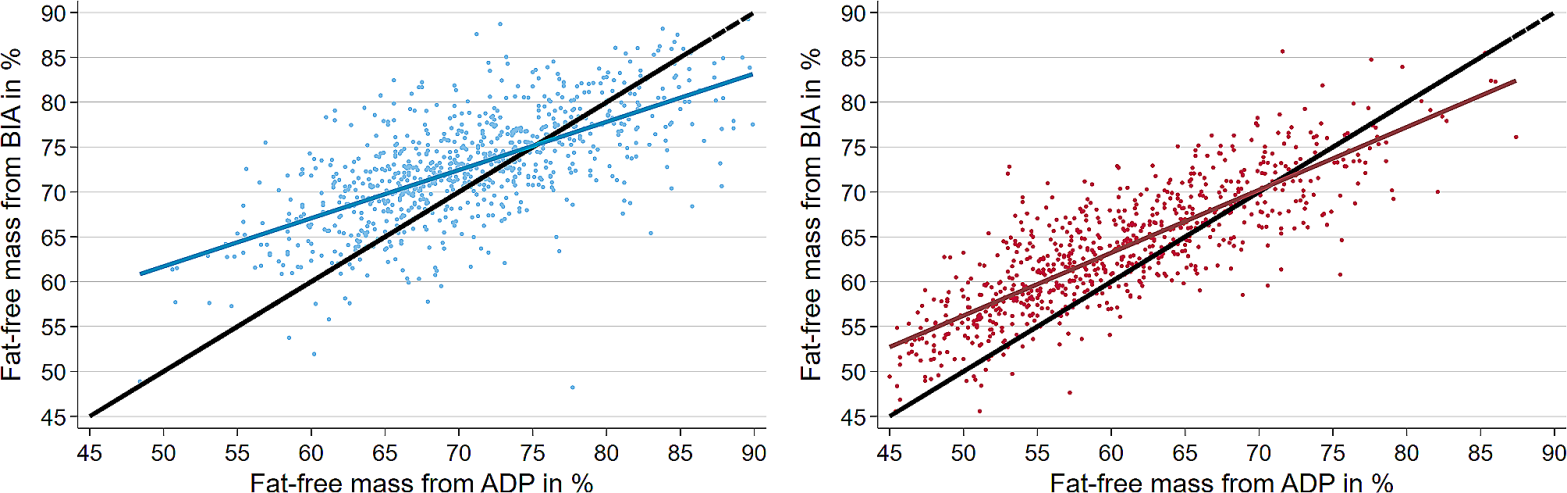
Association between ADP relative fat-free mass (FFM) and BIA FFM The models were stratified by sex (red = female, blue = male) and were adjusted for body height, age and time between ADP and BIA examination

Relative FFM_BIA_ (median = 68.7; inter-quartile range (IQR) = 62.5 to 74.0) was 3% higher than FFM_ADP_ (median = 65.7; IQR = 58.5 to 72.4). The median difference was more pronounced in females (63.3% vs. 59.6%) compared to males (72.7% vs. 70.3%). In linear regression analysis FFM_BIA_ and FFM_ADP_ were stronger associated in women compared to men (R^2^ = 0.70 vs. R^2^ = 0.43). Overall association expressed as R^2^ was 0.69 (Fig. 2). The correlation coefficients between FFM_BIA_ and FFM_ADP_ were 0.66 in men and 0.84 in women.

### Associations between anthropometric markers and FFM

**Fig. 3:**
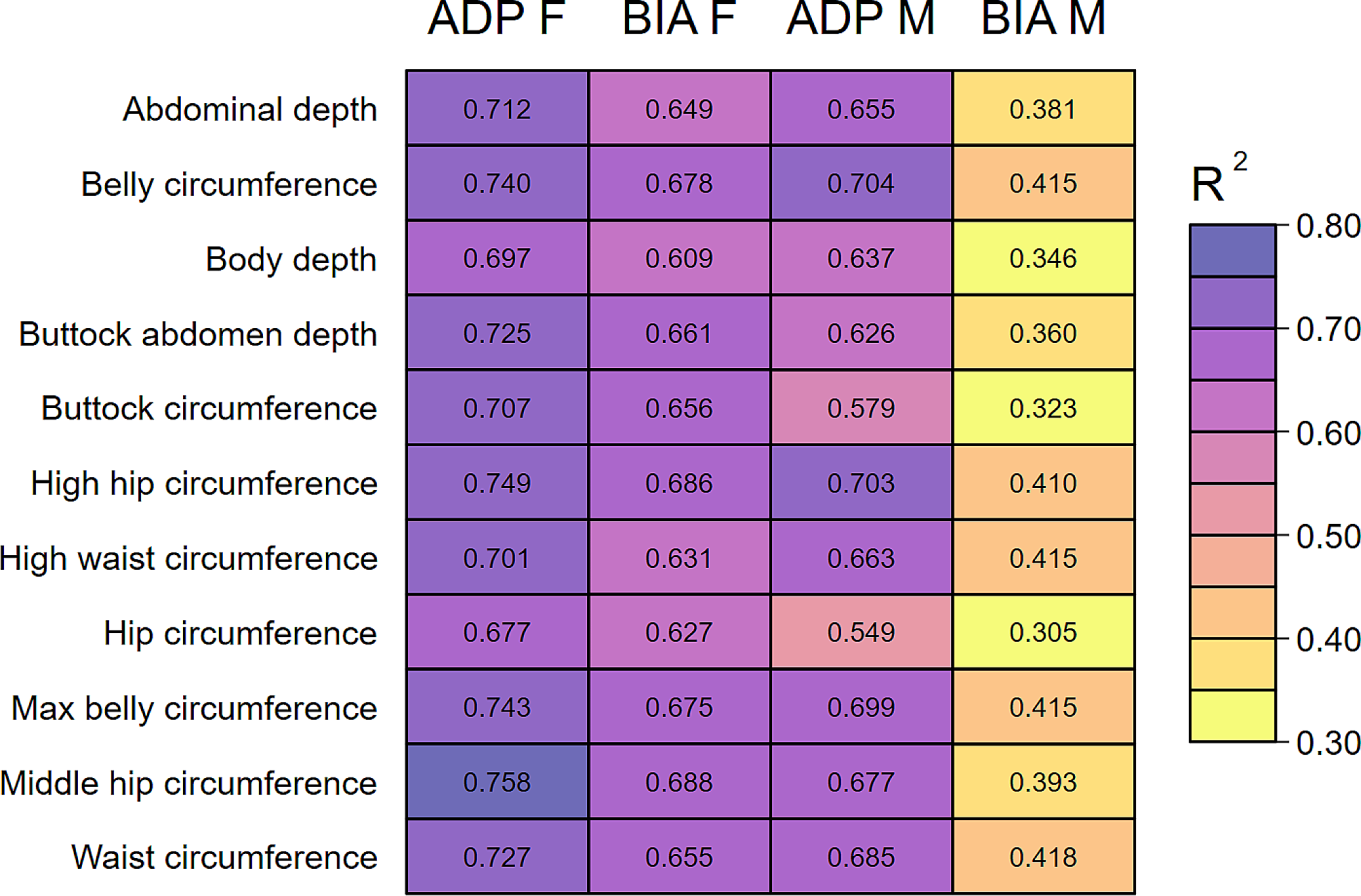
Body scan markers showing the strongest associations with relative fat free mass (FFM) Ranking according to the R^2^. Models were adjusted for body height, age (and time between core and BIA examination [for BIA FFM]). ADP F = FFM from ADP in females, BIA F = FFM from BIA in females, ADP M = FFM from ADP in males, BIA M = FFM from BIA in males

After adjustment for age and body height, the ten body scanner markers associated most strongly with FFM_ADP_ were very similar for men and women (Fig. 3). However, the order of these markers was slightly different and most of these markers were indicators of belly fat. Associations of the anthropometric markers with FFM_ADP_ were higher in women compared to men. The R^2^’s for the ten strongest markers for FFM_ADP_ ranged from 0.76 to 0.70 for women and from 0.70 to 0.58 for men. Calf and upper arm circumference, which are frequently used markers for FFM estimation in clinical practice, had considerably lower R^2^ values in both sexes and were not among the ten makers. The sex-specific associations of all body scan markers with FFM_ADP_ and FFM_BIA_ are listed in the Supplementary Tables 1 and 2. Accounting for multiple testing, all markers with a–log(p) > 6.84 were considered as statistically significant. With this threshold we observed 42 significant markers for FFM_ADP_ and 41 FFM_BIA_ in men. In women 42 significant markers were found for FFM_ADP_ for FFM_BIA_.

Correlations of body scan markers with FFM_BIA_ (adjusted for body height, age and time between core and BIA examination) were higher in women compared to men. Nonetheless, nine of the ten body scan markers showing the strongest association with FFM_BIA_ were the same in women and men. The body scan markers for belly fat, which were most tightly related to BIA, were also among the strongest markers for FFM_ADP_. The R^2^’s for the ten strongest markers for FFM_BIA_ ranged from 0.69 to 0.63 for women and from 0.42 to 0.32 for men.

**Fig. 4:**
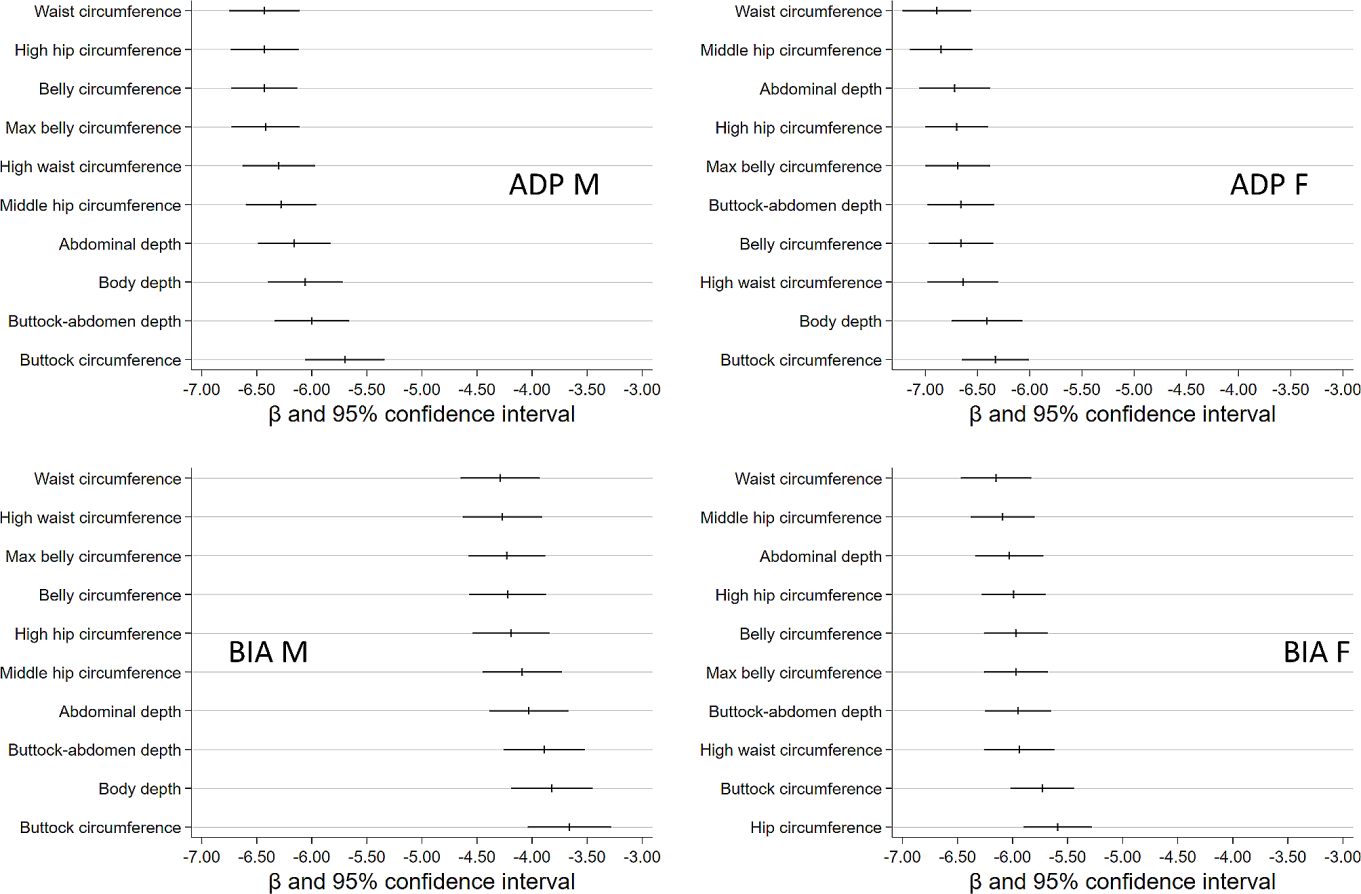
β-coefficients for the ten anthropometric markers most strongly associated with relative fat free mass (FFM) Models were adjusted for body height, age, (and time between core and BIA examination [for BIA FFM]). ADP M = FFM from ADP in males, BIA M = FFM from BIA in males, ADP F = FFM from ADP in females, BIA F = FFM from BIA in females

In females, the β-coefficients for the relation between anthropometric markers and FFM were very similar independent of measurement technique. In men, however, the β-coefficients were much smaller for FFM_BIA_ compared to FFM_ADP_ (Fig. 4). Independent of measurement technology and sex, the most strongly related anthropometric markers showed inverse relations with FFM.

Independent of measuring technique and sex, manually measured waist circumference showed a weaker association with FFM than waist circumference measured by body scanner. (Table 2) Manually measured hip circumference had a lower association with FFM than the automatically measured equivalents (“middle hip circumference” [MHC] and “high hip circumference” [HHC]) in females and males independent of measuring technique. Manually measured waist to hip ratio was only weakly associated with FFM independent of measurement technique and sex.

**Table 2:** Associations of manually measured anthropometric markers with relative fat free mass (FFM) adjusted for body height, age (and time between core and BIA examination [for BIA FFM])

	ADP F R^2^	BIA F R^2^	ADP M R^2^	BIA M R^2^
**Hip circumference**	0.738	0.683	0.593	0.346
**Waist circumference**	0.707	0.654	0.675	0.414
**Waist to hip ratio**	0.293	0.178	0.347	0.205

ADP F = FFM from ADP in females, BIA F = FFM from BIA in females, ADP M = FFM from ADP in males, BIA M = FFM from BIA in males

Further adjustment of the models for smoking status, alcohol consumption and the sports score did not change the results substantially. Exactly the same markers showed the strongest associations to FFM_BIA_ compared to FFM_ADP_ in a very slight different order.

**Fig. 5:**
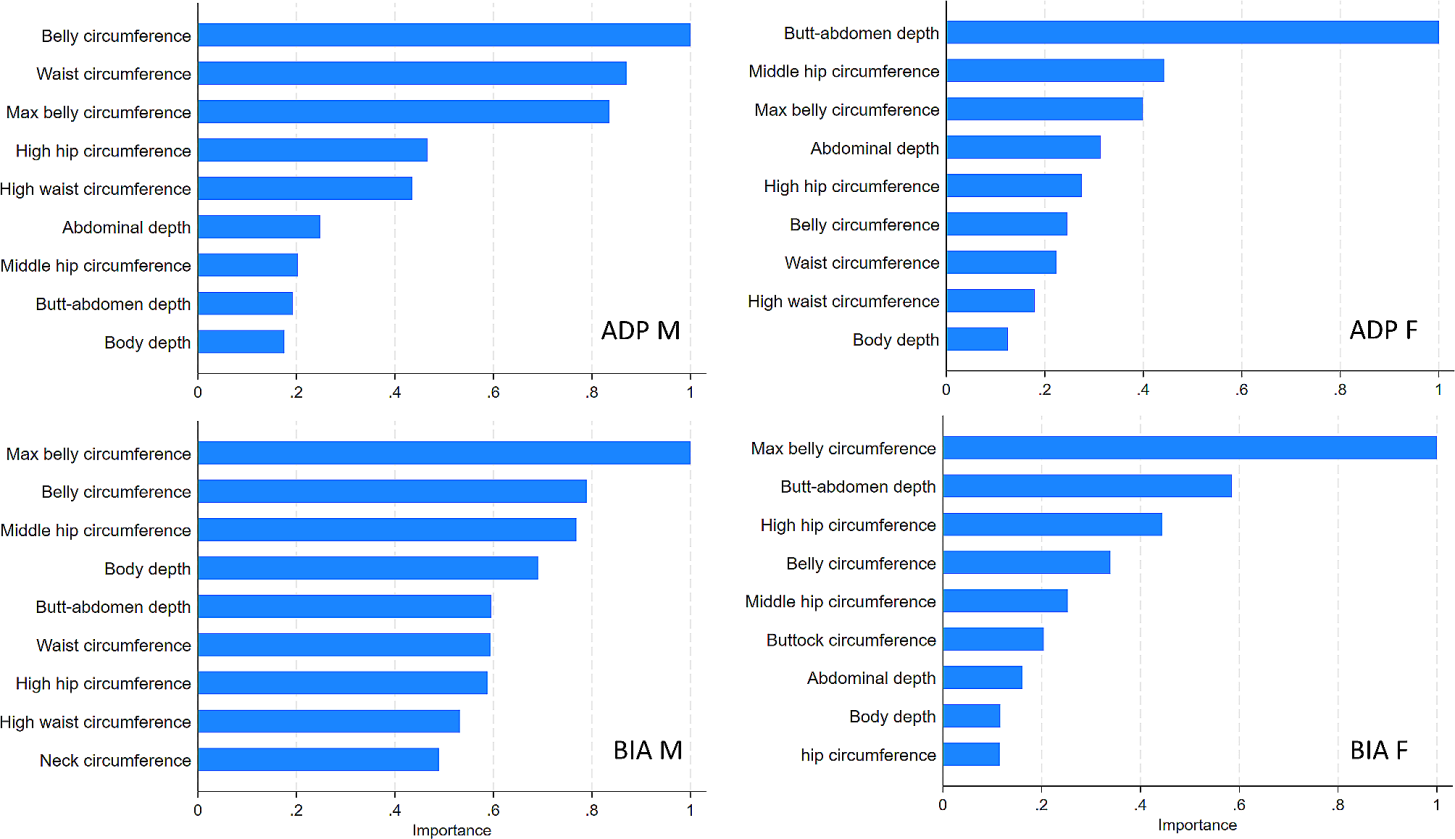
Importance ranking for body scan markers for relative fat free mass (FFM) as observed in random forest regression ADP M = FFM from ADP in males, BIA M = FFM from BIA in males, ADP F = FFM from ADP in females, BIA F = FFM from BIA in females

In sex-stratified random forest regression, the most important markers for FFM_ADP_ and FFM_BIA_ were similar to those detected in the logistic regression analyses and represented mainly markers of central adiposity (Fig. 5). The model specifications for the random forest models were as follows: FFM_ADP_ in men: numit = 300, numvars = 25, oob error = 3.32, root mean square error = 4.17; FFM_BIA_ in men: numit = 150, numvars = 8, oob error = 3.68, root mean square error = 4.82; FFM_ADP_ in women: numit = 150, numvars = 23, oob error = 2.96, root mean square error = 3.92; FFM_BIA_ in women: numit = 150, numvars = 20, oob error = 2.98, root mean square error = 3.78.

## Discussion

We investigated associations of anthropometric markers derived from an automated three-dimensional body scanner as well as manual measurement with FFM assessed by ADP and BIA in 1,593 individuals from Northeast Germany. We found strong inverse associations for markers of abdominal adiposity with relative FFM. Interestingly, effect estimates for markers derived automatically in the three-dimensional body scanner were much stronger compared to those measured manually. Furthermore, associations of body scan markers with FFM were stronger in women compared to men. With regards to measurement technique we found higher effect estimates for FFM_ADP_ compared to FFM_BIA_. Interestingly, our results highlight the potential to use of markers for central adiposity as a surrogate for FFM. This is especially surprising given that FFM is very much related to skeletal muscle mass and one would have hypothesized arm circumferences rather than markers of visceral adipose tissue [38].

By automatically collecting 47 different anthropometric sites using a three-dimensional body scanner, we were able to access a large, comprehensive set of anthropometric parameters. Importantly, the Global Leadership Initiative on Malnutrition (GLIM) recently endorsed anthropometric markers as alternatives to technology-based measurements of low muscle mass (i.e. low FFM), in settings where resources are limited [39]. We identified eleven anthropometric markers associated with FFM independent of technology. Hence, these parameters could be useful in the clinical assessment of FFM. These markers are generally considered to be surrogates of central adiposity: circumferences of belly, buttock, hip, waist, maximum belly, and depths of the abdomen and buttock. In current clinical practice the circumference of the arm and of the calf are used to estimate FFM. However, in our analysis these parameters only showed very weak associations. Thus, FFM may be related to fat accumulation in the waist, hip, and abdomen area of a person, regardless of sex or age. Since circumferences in this region can be taken relatively easy using an inelastic tape measure, the assessment will not depend on a large stationary device. We acknowledge that non-circumference anthropometric markers like “body depth” and “buttock abdomen depth” are not easily measured. The two reference points for applying the measuring tape are not on a horizontal plane. This suggests to use circumferences as convenient anthropometric markers in order to assess FFM in a clinical setting.

We show that automatically derived anthropometric markers had larger effect estimates compared to their manually measured equivalents. In addition, circumferences measured by scan were slightly different from those derived manually, which may be the result of different measuring positions. Assuming that parameters such as “high hip circumference” or “middle hip circumference” serve as the equivalent of manually measured, for instance, “hip circumference”, automatic body scanners may be superior to manual measurements or that the locations of the circumference of the automatic measurements contain more information for FFM prediction. Repp et al. [40] previously reported that the site of waist circumference measurement is important to improve the prediction of visceral adipose tissue. The exact measuring regions of aforementioned manually measured circumferences are described and defined in the ISO 7250-1:2017 standard [41]. Hence, future studies need to elucidate the most optimal site for circumference measurements.

Ng et al. [42] described three-dimensional scans as reliable methods to estimate FFM in healthy adults, albeit in a rather small study sample (*n* = 39). Bennett et al. [43] evaluated estimates of three-dimensional anthropometric markers and body composition (DXA scans). They reported strong association of body scanner anthropometrics and FFM based on DXA. Overall, previous studies already reported that anthropometrics may be used to estimate FFM, yet in relatively homogeneous study samples. The advantage of our study is that we included study participants representative of the general population. Hence, we extend the current knowledge by assessing the relation between anthropometrics and FFM in a very broad phenotype. We hope that this may help to promote the use of anthropometric markers as surrogates for FFM in clinical practice. This may be especially relevant for individuals living in rural or middle/low income regions with little access to technology. Health care professionals in these locations may use the identified anthropometric markers to monitor the individual response to specific treatments or interventions.

The beta-estimates for the associations between anthropometric parameters and FFM were depended on measurement technique. This was particularly seen in men, where the association between the anthropometric markers and FFM was much stronger for FFM_ADP_ than for FFM_BIA_. An explanation for this may be that the correlation between FFM_BIA_ and FFM_ADP_ was substantially higher in women compared to men. Previous studies reported that in individuals with obesity the different measurement techniques for FFM provide unequal results [44, 45]. In our study population men had a higher BMI compared to women. However, this difference may not explain a systematic bias in measuring FFM by BIA since previous studies also revealed conflicting results [46–49]. Our results identified very similar anthropometric parameters related to FFM yet with different effect estimates and in a different order for each sex. These findings may be related to sex specific cardiometabolic biomarkers [34], body fat distribution [35] and body composition [36].

In our study, the men had a higher prevalence of hypertension and type 2 diabetes mellitus compared to women. The presence of chronic diseases such as hypertension or type 2 diabetes are related to chronic inflammation or malnutrition resulting in a lower FFM. In addition, these metabolic conditions are associated with greater visceral fat [50, 51]. This again highlights the interesting nature of our results. In general, individuals with a greater BMI (i.e. more visceral adipose tissue) also have higher FFM, since locomotion of the greater body weight also requires more muscle mass. Yet, our findings highlight that when FFM is adjusted for FM and thus relative FFM is used, the statement above is not true. However, we believe that relative FFM is more important compared to absolute FFM with regards to health risk. Since chronic inflammation, malnutrition and aberrant fat distribution can cause altered hydration status and BIA equations depend on the consistency of hydration status [12, 52, 53], one may speculate that the observed differences in FFM_BIA_ and FFM_ADP_ are related to the hydration status of study participants with preexisting metabolic dysfunction.

This study has several strengths. We were able to assess a high number of anthropometric markers in a rather large study sample (*n* = 1,593). In addition, SHIP data uses very stringent data quality control. This is particularly related to standardization of non-invasive examination methods and data management [33]. FFM_BIA_ and FFM_ADP_ are established in clinical, as well as in research settings with a high degree of validity on a population level [14–16, 20, 21].

However, we were unable to use the gold standard for FFM measurement (DXA) due to radiation exposure and practicality. Our study population consisted exclusively of Caucasians from rural northeastern Germany, which supports validity within this ethnicity but limits conclusions about other study populations. In addition, we did not test for multicollinearity between our outcomes which could be considered a limitation. However, the manually measured anthropometric data may potentially not be as accurate as automatically scanned data, since an inter-observer bias cannot be excluded entirely [54]. Distinguishing whether there is a systematic difference between methods or a measurement area adjustment is critical to the association with FFM in order to transfer our results to an outpatient setting may be part of future research.

## Conclusion

Our findings suggest that anthropometric markers of visceral adiposity may be appropriate surrogates for relative FFM in the general population. The three-dimensional anthropometric markers were more strongly associated with FFM_ADP_ compared to FFM_BIA_ potentially related to the hydration status of individuals with metabolic dysfunction. Overall, our findings support the use of anthropometrics in daily clinical practice to estimate a person’s FFM for individualized therapy.

### Electronic supplementary material

Below is the link to the electronic supplementary material.

Supplementary Material 1

Supplementary Material 2

Supplementary Material 3

## Data Availability

Data from the “Study of Health of Pomerania” are available from the University Medicine Greifswald, Germany but restrictions apply to the availability of these data, which were used under license for the current study, and so are not publicly available. Data are, however, available upon reasonable request at https://transfer.ship-med.uni-greifswald.de/FAIRequest/ and with permission of the University Medicine Greifswald.
