# Supplementary material for: Associations between 47 anthropometric markers derived from a body scanner and relative fat-free mass in a population-based study
Source: BMC Public Health. 2024 Apr 18;24:1079. doi: 10.1186/s12889-024-18611-w (PMC11025281; doi:10.1186/s12889-024-18611-w)
Supplement: Supplementary file 3 — Supplementary Material 3 [file 12889_2024_18611_MOESM3_ESM.docx]

Supplementary Material and Methods

Extended description of the measurements from 3DO body scanner, bioelectrical impedance analysis and air displacement plethysmography

3DO body scan

Anthropometric data was measured with a 3D body scanner *(VITUS Smart XXL, Vitronic, Wiesbaden, Germany)* driven by the software *AnthroScan Professional (Version 3.0.7, Human Solutions GmbH, Kaiserslautern, Germany).* This measuring technique is based on the optical triangulation process using four lasers and eight cameras, to date one of the most precise method of contactless three-dimensional capture of body shape. The apparatus allows the observer to receive deformation-free measurements, since neither the lasers nor the sensors enter into any physical contact with the subject^1^.

The measurement provides a detailed 3-dimensional image of the participants’ body surface and extracts up to 150 different standard anthropometric markers, including circumferences, lengths, distances, areas, volumes and their ratios within 10-15 seconds.

Participants wore underwear while being scanned and also wore a fabric head cap in order to reduce measurement error on account of the volume of their hair.

A total of 3 scans were accomplished: ISO-scan in sitting position, ISO-scan in standing position, and standard scan.

After the second scan, participants were asked to step out of the body scanner for manual somatometry measurement of waist and hip circumference. After that, 5 hemispherical shaped (diameter 2cm) markers (physical markers) were attached to defined points on the participant’s left anterior part of the body: at the level of the bottom rib, at the level of manual waist circumference measurement, at the level of the iliac crest, at the level of manual hip circumference measurement, at the level of the greater trochanter, respectively. In that process, it is important to place the markers in correct height; a lateral offset is harmless and, in many cases, necessary. To be able to clearly identify the markers in the scan, two markers must be at least 4 cm apart from each other. To avoid also problems with the shadow of the arms (especially with obese individuals), the markers cannot be attached directly to the side of the test person, but to the left anterior side of the body. Extraction (reading) of body measures from the ISO-Scans was performed on the basis of ISO 7250-1:2010^2^.

*ISO-scan in sitting position*: participants were asked

- to sit on the scan chair
- the chair is adjusted to the body size, knee angle 90° in sitting position with ground-touching feet
- thighs are completely resting on the chair
- to sit up straight ("spread out") - allow upper arms to hang down freely
- to hold the right forearm at a right angle horizontally forward, the right hand holds a measuring stick vertically
- to hold the left forearm at a right angle horizontally forward, stretch out the left hand with closed fingers
- to hold the head horizontally and looking forward (Frankfurt horizontal plane)
- to continue breathing normally

*ISO-scan in standing position*: participants were asked

- to stand on the measuring platform
- to stand with the feet closed
- to stand upright ("spread out")
- to let the left arm hang down freely, the left hand holds the measuring device at the sagittal plane with horizontal grip axis
- to let the right upper arm hang down relaxed
- to hold the right forearm horizontally forward at right angle, to hold the hand as a relaxed fist
- to keep the head in a horizontal position facing forward (Frankfurt horizontal plane)
- to continue breathing normally

*Standard scan*: for investigation of general body composition, such as height and several lengths and circumferences, participants were asked

- to stand on the measuring platform
- to place the feet on the foot markers and, depending on the width of the shoulders, to reduce or increase the distance between the feet (instructions given by the examiner)
- to stand upright
- to clench the hands to his fist and keep them parallel to the body at the level of the trouser seam
- to stretch the arms slightly away from the body and bend them (instructions: carrying heavy suitcases)
- to keep the head horizontal and facing forward (Frankfurt horizontal plane)
- to continue breathing normally

The room was darkened and the measuring chamber was closed except for a gap. This allowed the examiner to supervise the scan posture of the test person. In the prepared Scan-Wizard the respective scan was started.

Bioelectrical impedance analysis

BIA is performed using a multifrequency *Nutriguard-M* device *(Data Input GmbH, Pöcking, Germany)* and the *NutriPlus* software *(Version 5.4.1, Data Input GmbH, Pöcking, Germany)*. R (resistance) and X_c_ (reactance) are measured applying electric currents of 800mA at 5, 50, and 100 kHz following the manufacturer’s instructions^3-5^. Source and sensor electrodes are placed on the dorsum of hand, wrist, ankle and dorsum of foot of the dominant body side^6^.

Subjects with pacemakers did not take part in the BIA. In addition, the following guidelines were adhered to, otherwise the results of the BIA could have been falsified:

- the subject was fasting for the last 4-5 hours
- the subject's last sporting activity was at least 12 hours ago
- the subject's last alcohol consumption was at least 24 hours ago
- the extremities had a normal temperature
- the room temperature was at least 22-26°C
- Right before measurement, participants were asked:
- to lay in supine position on a simple examination couch
- to rest like this for at least 10 minutes before the start of measurement
- to have their legs spread out at a 45° angle
- to spread out the upper extremities at a 30° angle (no body contact)
- to have no part of the body in contact with external metal objects
- to not hold any objects in their hand (e.g. handkerchief)

Likewise, the subject's extremities were supposed to lie at body height when measured. If an arm or leg was positioned lower or higher during the measurement, this could have falsified the measurement results.

The BIA was carried out on the dominant side of the subject (NOTE: “dominant hand” was defined as “the hand that the respondent would use to cut with scissors or to cut bread with a knife”). If this was right-handed, the electrodes were attached to the hand and foot of the right-hand side of the subject. Therefore, before the examination, the examiner had to ask which was the “strong” hand of the test person. If the test person indicated that he was ambidextrous, measurements were taken on the right-hand side of the test person. It was also important that the electrodes on the hand and foot were both placed on the same side. The electrodes were applied to dry and oil-free skin. It was therefore important to always clean and dry the skin on both the wrists and ankles with alcohol before the examination. If the skin was very dry, it was beneficial to apply ECG paste. Electrodes were only used once.

The tetrapolar and ipsilateral measurement with adhesive electrodes has established itself worldwide as a method for medical bio impedance measurement, which is why 2 joint electrodes were attached to the hand and foot of the same side of the body. The precise positioning of the electrodes is particularly important, since a deviating placement of the measuring electrodes of only one centimeter can lead to a measuring deviation of up to 20 ohms, for example.

The four electrodes were attached to the hand and foot as follows (for more details, see supplement, SOP Bioelectric impedance analysis):

*Wrist electrode (measuring electrode):* Locate the highest point of the ulnar head. At this point, draw a horizontal line across the wrist. Glue the proximal (inner) edge of the electrode along this line. The electrode should mainly be located above the soft, more conductive joint gap. Attach the black alligator clip of the hand cable (yellow sleeve) to the tab of the electrode.

*Finger joint electrode (signal electrode):* Identify the highest points of the 2^nd^ and 3^rd^ finger base joint. Draw a horizontal line across these joints through these points. Glue the distal (outer) edge of the electrode along this line. The main surface of the electrode should lie over the soft tissue between the 2^nd^ and 3^rd^ metacarpals. Attach the red alligator clip of the hand cable (yellow sleeve) to the tab of the electrode.

*Ankle electrode (measuring electrode):* Identify the highest points of the outer and inner ankles. Draw a horizontal line through these points across the dorsum of the foot. Glue the proximal (inner) edge of the electrode along this line. The main surface of the electrode should lie above the joint gap of the upper ankle. Attach the black crocodile clip of the foot cable (red sleeve) to the tab of the electrode.

*Toe joint electrode (signal electrode):* Identify the highest points of the 2^nd^ and 3^rd^ toe base joint (bend toes so that the joint is clearly visible). Draw a horizontal line across these joints through these points. Glue the distal (outer) edge of the electrode along this line. The main surface of the electrode should lie over the soft tissue between the 2^nd^ and 3^rd^ metatarsals. Attach the red crocodile clip of the foot cable (red sleeve) to the tab of the electrode.

After the electrodes had been adjusted, weak currents were passed through the body through the electrodes twice and body resistances and phase angles were measured.

Air displacement plethysmography

Air displacement plethysmography was performed using a *BOD POD^®^* *(COSMED Deutschland GmbH, Werneck, Germany)* device according to the manufacturer’s recommendation *(COSMED Deutschland GmbH, Werneck, Germany)*.

The used method is a densitometric measuring method and can be used to determine body volume, body density and lung volume, fat mass and FFM. The body weight was determined during the examination with an electronic scale integrated into the BOD POD device. Participants were only wearing relatively tight-fitting underwear and socks during examination. They were asked to sit in the BOD POD measuring chamber in a relaxed position but not leaning back. Respiration should be normal and the subjects wore a swimming cap. Afterwards the first measurement was conducted. After a series of measurements according to the manufacturer the door of the BOD POD could be opened and the subjects could leave the device.

1. Bretschneider T, Koop U, Schreiner V, et al. Validation of the body scanner as a measuring tool for a rapid quantification of body shape. Skin Res Technol. 2009;15(3):364-369.

2. ISO. ISO 7250-1:2017 - Basic human body measurements for technical design. Switzerland 2017.

3. Kyle UG, Bosaeus I, De Lorenzo AD, et al. Bioelectrical impedance analysis--part I: review of principles and methods. Clin Nutr. 2004;23(5):1226-1243.

4. Kusztal M, Kleszczynski J, Weyde W, et al. Pulse volume changes recorded by air plethysmography during single hemodialysis sessions. Blood Purif. 2008;26(6):498-504.

5. GmbH D-I. Das B.I.A.-Kompendium 3. Ausgabe. Darmstadt 2005.

6. Völzke H, Alte D, Schmidt CO, et al. Cohort profile: the study of health in Pomerania. Int J Epidemiol. 2011;40(2):294-307.
